# EGFR‐KDD Myofibroblastic Neoplasm or Congenital Peribronchial Myofibroblastic Tumor (CPMT)? Report of a Congenital Myofibroblastic Neoplasm With Unusual Histologic Features

**DOI:** 10.1002/gcc.70032

**Published:** 2025-04-03

**Authors:** Emma Rullo, Sabina Barresi, Sabrina Rossi, Sara Patrizi, Evelina Miele, Marta Barisella, Michela Casanova, Andrea Ferrari, Stefano Chiaravalli, Gloria Pelizzo, Rita Alaggio

**Affiliations:** ^1^ Pathology Unit, Bambino Gesù Children's Hospital Rome Italy; ^2^ Department of Molecular Medicine Sapienza University Rome Italy; ^3^ Onco‐Hematology, Cell Therapy, Gene Therapies and Hemopoietic Transplant Bambino Gesù Children's Hospital and Research Institute Rome Italy; ^4^ Pathology Unit, ASST Fatebenefratelli‐Sacco Milan Italy; ^5^ Pediatric Oncology Unit, Fondazione IRCCS Istituto Nazionale Tumori Milan Italy; ^6^ Department of Oncology and Hemato‐Oncology University of Milan Milan Italy; ^7^ Department of Pediatric Surgery, Children's Hospital “Vittore Buzzi” Milan Italy; ^8^ Department of Biomedical and Clinical Sciences “L. Sacco” University of Milano Milan Italy; ^9^ Department of Medico‐Surgical Sciences and Biotechnologies Sapienza University Rome Italy

**Keywords:** congenital, EGFR‐KDD, infantile fibrosarcoma, lung tumor, myofibroblastic tumor, NGS, pediatric

## Abstract

EGFR‐kinase‐domain duplication (KDD) has been reported in Infantile fibrosarcoma‐like myofibroblastic tumors and cellular mesoblastic nephroma. We report a pulmonary neoplasm with EGFR‐(KDD) and infantile fibrosarcoma‐like histologic features in a female infant with an unusual clinical and histologic evolution, characterized by persistent disease with morphologic features of Congenital Peribronchial Myofibroblastic Tumor (CPMT) after chemotherapy and targeted therapy. The CPMT morphology with *EGFR‐KDD* in the post‐therapy specimen might be an evolution induced by the treatment, which suggests the hypothesis that CPMT is part of the morphologic spectrum of infantile fibrosarcoma/cellular mesoblastic nephroma.

## 1 Introduction

Congenital peribronchial myofibroblastic tumor (CPMT) is recognized by the Pediatric WHO classification, 5th edition, as an extremely rare myofibroblastic neoplasm of the lung, with less than 24 cases reported so far, all developing in utero or during infancy [1, 2, 3, 4, 5, 6, 7, 8, 9, 10, 11, 12, 13, 14, 15, 16, 17, 18, 19, 20, 21] (Table 1). CPMT is characterized by histologically bland myofibroblasts arranged in intersecting fascicles with a benign‐appearing cartilage component. The potential relationship of CPMT with infantile fibrosarcoma (IF) has been raised by the identification of *ETV6*::*NTRK3* fusion in three cases [1] and, more recently, of a kinase‐domain duplication of *EGFR* (*EGFR*‐*KDD*) in four cases [21]. However, its favorable prognosis has been considered a strong argument in favor of its completely benign nature, different from IF. Interestingly, *EGFR‐KDD* was initially identified in classic or mixed variants of congenital mesoblastic nephroma (CMN), which are composed of myofibroblasts in fascicular pattern and may display nests of benign cartilage. In mixed CMN areas with IF‐like features are seen. Cellular CMN represents the renal counterpart of IF and shares with IF the *ETV6::NTRK3* fusion or the other NTRK rearrangements reported in IF. By contrast, *EGFR‐KDD* is rare in cellular CMN. Similarly, *EGFR*‐*KDDs* have been detected in extra‐renal mesenchymal neoplasms with histologic features reminiscent of mixed CMN or IF/cellular CMN.

We report a unique myofibroblastic neoplasm with *EGFR*‐*KDD* in a female newborn. The CPMT histology in posttreatment residual disease further supports the potential relationship with CMN [21].

## 2 Materials and Methods

### 2.1 Immunohistochemistry

Immunohistochemistry was performed on the Dako Omnis platform (Agilent) according to the standard protocols, using the following primary antibodies: Smooth Muscle Actin (SMA), S100, CD34, SALL4, Desmin, Myogenin, Cyclin D1, BCOR, SOX10, CK MNF116, alpha‐fetoprotein, and H3K27me3.

### 2.2 RNA Analyses

RNA was extracted from 5‐μm FFPE sections using the Maxwell CSC FFPE DNA and RNA Extraction Kit (Promega) for the automatic extraction.

To identify genomic rearrangement, 200 ng of RNA were used for library preparation with the Archer Custom Fusion Plex Kit (ArcherDX, CO), according to the manufacturer's protocols. NGS data were analyzed using Archer Data Analysis Software v6.2.3.

### 2.3 Methylation Profile Analysis

DNA was extracted from FFPE sections and analyzed with the Human MethylationEPIC v2.0 BeadChip arrays (Illumina), according to the manufacturer's instructions. Raw .idat files were then uploaded to the online platforms DKFZ Sarcoma Classifier v12.3 [22] and to the EPIDIP (Epigenomic Digital Pathology) server. Data were further processed as previously described [23] to carry out t‐distributed stochastic neighbor embedding (t‐SNE) analysis.

## 3 Case Report

**FIGURE 1:**
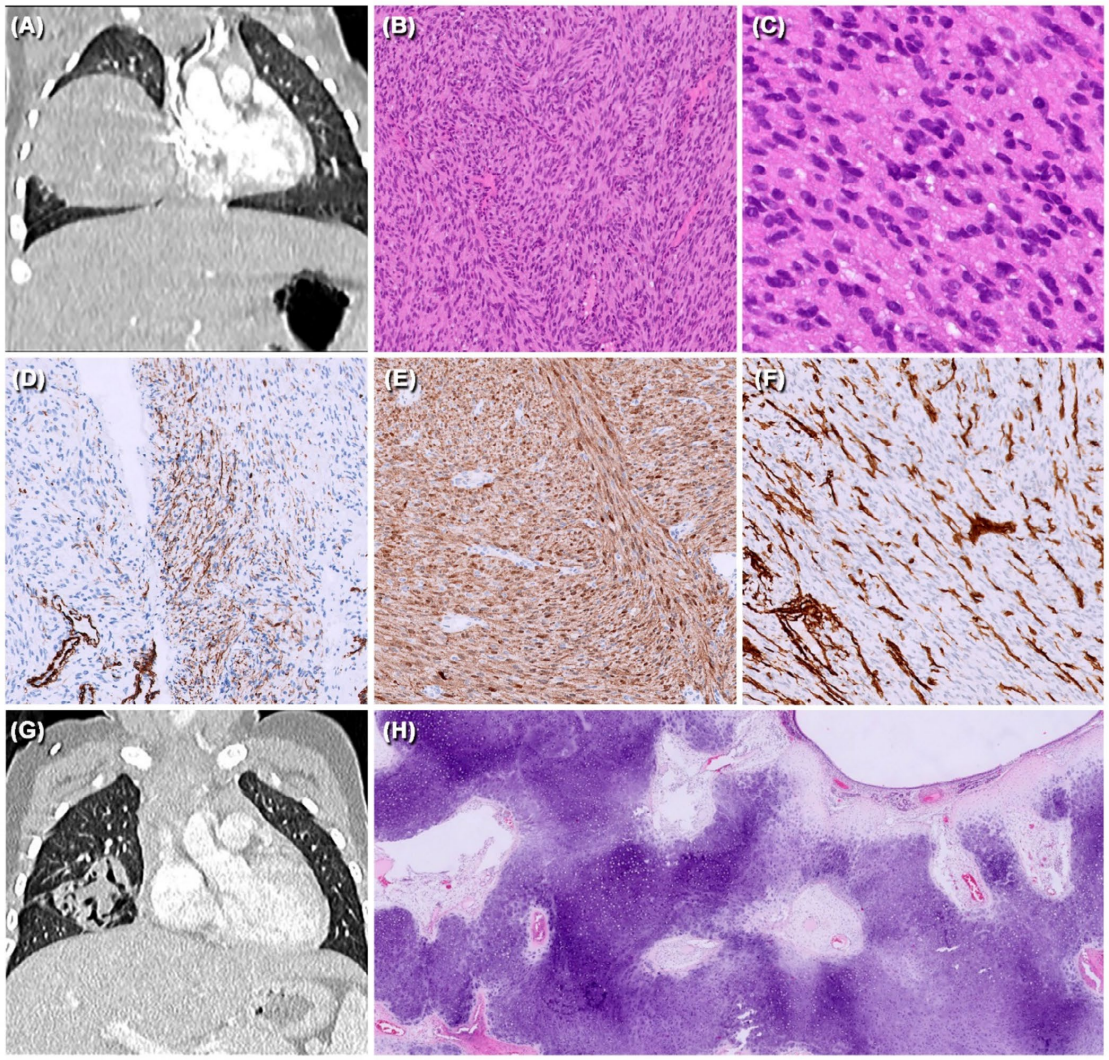
(A) MRI showing a 5 cm mass dislocating mediastinal structures; (B) low and (C) high power magnification of the biopsy sample: A spindle cell proliferation made of elements having round to oval nuclei, eosinophilic cytoplasm with undefined cell borders and arranged in a vaguely fascicular pattern is evident; (D) SMA was focally positive in a minority of cells; (E) S100 was diffusely positive; (F) CD34 was negative; (G) radiological appearance of the lesion after the combined therapy just before surgery; (H) examination of the surgical specimen after therapy revealed a complete replacement of the spindle cell proliferation by islands of mature cartilage.

A 4‐day‐old female developed respiratory distress requiring oxygen therapy. Chest radiography revealed a hilar mass measuring 5 cm as the maximum dimension within the right hemithorax, with mediastinal structures dislocation (Figure 1A). Prenatal history was silent, with negative imaging except for polyhydramnios at 32 WG. An incisional lung biopsy was performed with a diagnosis of IF‐like myofibroblastic neoplasm harboring *EGFR*‐*KDD* (exons 18–25) based on histology and molecular findings. While molecular studies were performed, a spontaneous decrease in the volume of the lesion was observed, which continued in the following months. However, at 5 months from diagnosis, a follow‐up magnetic resonance imaging (MRI) scan showed disease progression. The patient underwent chemotherapy with vincristine and actinomycin‐D (VA regimen), and subsequently, ifosfamide was added due to lack of response. Despite the introduction of the alkylating agent, the disease progressed after 6 months of conventional chemotherapy, and therefore, the vinorelbine and Nimotuzumab combination was started on a compassionate use basis. The new combination, given weekly for 12 weeks and then every 2 weeks for 6 months, achieved a partial remission. The bronchi, previously entrapped within the lesion although more discernible, appeared ectasic and tortuous (Figure 1G). Thus, the surgical resection of the right medium and lower lobe was performed. The patient is alive without disease 7 months after surgery.

**TABLE 1:** Clinicopathological characteristics and immunophenotype of the previously reported cases of CPMT.

Ref	Age (weeks*)	Sex	Mitoses	Immunophenotype
[2]	3	M	1–5/10 HPF	Vimentin+, Desmin−/+ (rare cells), SMA−/+ (< 5%), S100+/− (near cartilage)
[3]	35	F	0–3/10 HPF	Vimentin+, SMA+/−, Desmin−, S100−, GFAP−, alpha‐fetoprotein—
[4]	25	M	0‐4/HPF	Desmin−, SMA−, S100−, CK−
[5]	29	M	NA	Calponin+, CD31−, CD34−, S100−, SMA−
[6]	24	M	Frequent but not abnormal	Vimentin+, HHF35+, SMA+/−, CD34−, CD99−
[7]	23	M	Brisk	Vimentin+, SMA+, Myogenin−, Desmin−, MSA−, CD117, S100−, CD34− alpha1‐antitrypsin
[8]	4 (post‐natal)	F	8/10 HPF	Vimentin+, SMA+, Desmin−, EMA−, CK−, factor VIII−, S100−
[9]	32	F	5/10 HPF	SMA+ (weak), Desmin−, OCT4+
[9]	0	F	Rare	SMA+ (strong), Desmin+/−, OCT4−
[10]	30	M	NA	Desmin+
[11]	27	M	10/10 HPF	Vimentin+, SMA+/−, CD34+/−, Desmin+/−, MSA+/−, S100+/− (near cartilage), CK AE1/AE3−, EMA−, ALK1−, H‐Caldesmon−, Calponin−, EBER‐1—
[12]	35	M	4–5/10 HPF	Vimentin+, SMA−, Desmin‐, S100−, CD34−, HHV‐8−, ALK1−, alpha‐fetoprotein−, NSE—
[13]	27	M	NA	Vimentin+, SMA+/−, CD68+/−, CD31−, CD34−, S100−, Desmin−
[14]	8 (post‐natal)	M	Frequent	SMA−, Desmin−, Myogenin−, CD34−, CK AE1/AE3−
[15]	28	M	NA	Vimentin+, SMA+, S100+, CD99+, p53+, H‐Caldesmon−, Desmin−, CD34−
[16]	33	F	NA	NA
[17]	7	M	1/10 HPF	Vimentin+, SMA+, S100+, CD117−, ALK1−, Desmin−, Myogenin−, MyoD1−, ROS1−, CD34
[18]	30	F	1/10 HPF	SMA+/−, S100−, CD34−, CK−, EMA−, ALK1−, Desmin−, OCT3/4—
[19]	3,5 (post‐natal)	M	3‐5/HPF	Vimentin+, SMA+, Calponin, CD99+, S100+ (weak), SOX10−, CD34−, ALK1−, EMA−, CK−, pan‐TRK−, STAT6−, Desmina−, MyoD1−
[20]	31	NA	NA	Vimentin+, SMA+ (weak), Desmin−, CD34‐, CK AE1/AE3, TTF‐1−, HHF‐35−, S100+/− near the cartilage
[21]	0 (post‐natal)	M	Rare	SMA+ (strong in perivascular spindle cells, weak next to cartilage), S100+/− (patchy)
[21]	20	M	Brisk, rarely atypical	SMA+ (weak in spindle cells)
[21]	8 (post‐natal)	M	Frequent	SMA+ (weak in spindle cells), S100− (positive in cartilage)
[21]	20	F	Focally high, sometimes atypical	NA

Abbreviation: NA: not applicable.

*Refer to gestational weeks unless otherwise specified.

### 3.1 Histology

Histological examination of the initial biopsy revealed a densely cellular lesion, composed of fascicles of elongated cells with round to oval nuclei, indistinct cell borders, and eosinophilic, focally vacuolated cytoplasm (Figure 1B,C). Alveolar spaces were entrapped. Mitoses were sparse. Necrosis was absent.

Immunohistochemistry demonstrated positive immunostaining for SMA (focal), S100 (diffuse), and Cyclin D1 (Figure 1). CD34, Desmin, Myogenin, SALL4, CK MNF116, BCOR, SOX10, and alpha‐fetoprotein were negative (Figure 1). Expression of H3K27me3 was retained.

At surgical excision, the lobectomy specimen showed a multilobulated translucent nodule with firm consistency encircling the bronchial wall. Histologically, the lesion was represented by well‐demarcated islands of mature cartilage forming peribronchial cuff‐like structures of variable size, surrounded by fibro‐edematous and vascular stroma (Figure 1H). A complete histologic evaluation of the entire lesion did not demonstrate other components.

The overall histological features demonstrated a morphologic variation, from a benign myofibroblastic neoplasm at initial diagnosis to a typical CPMT in the posttreatment resection specimen. The remaining lung parenchyma was unremarkable except for scattered areas of atelectasis.

### 3.2 Molecular Analyses

NGS analysis demonstrated an *EGFR‐KDD* (exons 18–25) in both diagnostic biopsy and surgical excision (Figure S1). Alterations involving the *DICER1* gene that could be suspected considering the presence of cartilage within the post‐therapy specimen were not found. Methylation profile analysis performed on the posttreatment surgical specimen showed no matching with the methylation classes of the Heidelberg sarcoma classifier. In adjunct, the tumor did not cluster with the previously reported *EGFR*‐*KDDs* myofibroblastic neoplasms [23].

## 4 Discussion

CPMT is a rare congenital/infantile neoplasm of the lung, firstly described by McGinnis in 1993 [2]. Tumors with similar morphology had been previously diagnosed as fibrosarcoma, leiomyosarcoma, mesenchymal malformation, or hamartoma, according to the interpretation and relevance given to the histologic components. CPMT is considered a distinctive entity despite the morphological similarity of its spindle cell component to IF, because of its favorable clinical outcome and peculiar morphology. The evidence of *ETV6*::*NTRK3* fusion documented in at least three cases [1] further reinforced the hypothesis of a relationship with IF [11]; however, the lack of histologic documentation represented an important limitation [1, 9, 11]. Alobeid et al. reported a complex rearrangement involving chromosomes 4, 8, and 10 [3]. Another case was found to have both a *JAK2* and a *SMO* mutation by NGS analysis [18]. More recently, *EGFR‐KDD* has been identified in a series of 4 CPMTs, which showed a spindle cell component histologically similar to the initial biopsy of the current case, besides variable amounts of cartilage [21].

In fact, in the current case, the initial open biopsy appeared as a histologically bland proliferation of spindle cells with eosinophilic cytoplasm in a vaguely fascicular pattern. The morphology was consistent with previously reported *EGFR‐KDD* myofibroblastic neoplasms of soft tissue [23, 24]. However, in spite of the large size of the initial biopsy and its resemblance to the spindle cell component of CPMT, cartilage was not seen. By contrast, the residual posttherapy neoplasm was composed of mature cartilage nests in a characteristic peribronchial cuff‐like arrangement typical of CPMT.

To the best of our knowledge, this is the first *EGFR‐KDD* pediatric mesenchymal neoplasm treated by a combination of conventional CT and targeted therapy. A volume reduction was obtained with a combined therapy including Nimotuzumab, an anti‐EGFR monoclonal antibody targeting the specific molecular alteration. Whether the morphologic evolution is a maturation process induced by the targeted treatment or is the expression of the biologic potential of the specific tumor type, that is a CPMT, is debatable. However, the latest possibility is supported by the evidence of histological evolution and even spontaneous regression in CPMT [7, 15]. A prenatally diagnosed CPMT spontaneously regressed before birth, while another CPMT demonstrated a decrease of cellularity and mitoses in favor of the cartilaginous component in a time span of 4 months [7, 15]. Chang et al. suggest a “maturational” evolution in one of two CPMTs based on higher cellularity, more frequent mitoses, and less mature cartilage in the tumor from the youngest patient [9]. Similarly, in the recent series by Younes et al., a fetal biopsy of a CPMT (Case 3) was composed of primitive, mitotically active spindle cells with only sparse immature cartilage, whereas in the postnatal excision, the neoplasm was predominantly cartilaginous, with less frequent mitoses [21]. Although in our case the targeted treatment was effective in reducing the tumor volume, the persistence of cartilage suggests that the use of an anti‐EGFR monoclonal antibody, as proposed by Younes et al. for unresectable tumors, might be of limited efficacy in CPMT. In addition, the evidence in our case of *EGFR*‐*KDD* in the cartilage demonstrates that it is neoplastic and clonally derived from the spindle cell component.

The recurrence of *EGFR‐KDD* in 5 CPMT, including also our case, strongly supports its role as a driver molecular alteration. As suggested by Younes et al., *EGFR*‐*KDD* might play a role in keeping an activated EGFR status, known to be involved in lung development during fetal life. The authors also suggest a potential relationship with mixed and classic mesoblastic nephroma, both carrying *EGFR*‐*KDDs*, while it is found very rarely in cellular mesoblastic nephroma, which is considered the renal counterpart of IF, characterized by *ETV6*::*NTRK3* fusion.

While the histologic border between classic mesoblastic nephroma and cellular mesoblastic nephroma is well defined and supported by the absence of *ETV6*::*NTRK3* fusion or other *NTRK* rearrangements in classic mesoblastic nephroma, mixed CMN represents a “gray zone” with histologic overlap with both cellular and classic CMN. Thus it is not surprising the identification of *EGFR‐KDD* in a subset of cellular CMN. This is also paralleled by the evidence in *EGFR*‐*KDD* myofibroblastic neoplasms in soft tissue of lesions with hybrid features of benign myofibroblastic lesions and more densely cellular areas, similar to mixed CMN and others with IF‐like features or even lipofibromatosis‐like [21, 24, 25].

Thus, while *ETV6*::*NTRK3* fusion identifies the homogeneous group of IF and the renal counterpart (cellular or mixed CMN), *EGFR‐KDD* mesenchymal neoplasms are a heterogeneous group of mesenchymal neoplasms which deserve further investigation. However, the few cases reported as CMN in the kidney, CPMT in the lung and as IF‐like or hybrid lesions in soft tissue appear to have a benign clinical course.

It may be speculated that cartilage is seen in CPMT and CMN because of the organ‐specific multipotential ability of primitive mesenchymal cells. In fact, peribronchial mesenchymal cells are involved in bronchial cartilage development. Although the mature kidney does not show cartilage, the latter is frequently found in renal dysplasia. In addition, as recently proposed by Younes et al., the infiltrative pattern of both CPMT's and CMN's might reflect the early neoplastic transformation of these mesenchymal cells, with a tumor growth in tandem with the organs during fetal life [21].

Methylation profiling may be a useful tool to define the cell of origin and the histogenetic relationship of neoplasms. In this case, it was not conclusive; however, the analysis had been carried out only on the post‐therapy specimen, which might be a limitation. The lesion did not cluster with other *EGFR*‐*KDD* neoplasms of soft tissue previously reported [23]. Although a larger number of cases are needed to explore this potential relationship, this finding might reflect a more terminally differentiated phenotype towards cartilage in CPMT.

In conclusion, we reported a distinctive congenital neoplasm of the lung with *EGFR*‐*KDD* and histologic features of CPMT, treated by a combination of CT and targeted therapy. The morphologic evolution is in keeping with previously described cases and highlights the neoplastic nature of the spindle cell component, which “matures” into cartilage. This is supported by the evidence of *EGFR*‐*KDD* in the cartilaginous residual disease [2, 6, 7]. The evidence of *EGFR‐KDD* corroborates the recent identification of this alteration as a molecular driver in CPMT, further supporting its potential relationship with CMN and the rare myofibroblastic neoplasms of soft tissue with this molecular alteration.

## Ethics Statement

Ethical approval is not required as this is a case report. All the clinical and pathologic investigations detailed in the manuscript have been conducted in accordance with the Declaration of Helsinki and its later amendments.

## Consent

Informed consent for publication of data and images was obtained from the parents as the subject of the report is a minor.

## Conflicts of Interest

The authors declare no conflicts of interest.

## Supporting information


**Figure S1.** Supporting Information.

## Data Availability

The data that support the findings of this study are available from the corresponding author upon reasonable request.
