# Supplementary material for: EGFR‐KDD Myofibroblastic Neoplasm or Congenital Peribronchial Myofibroblastic Tumor (CPMT)? Report of a Congenital Myofibroblastic Neoplasm With Unusual Histologic Features
Source: Genes Chromosomes Cancer. 2025 Apr 3;64(4):e70032. doi: 10.1002/gcc.70032 (PMC11967378; doi:10.1002/gcc.70032)
Supplement: Supplementary file 1 — Figure S1. Supporting Information. [file GCC-64-e70032-s001.pptx]

## Slide 1
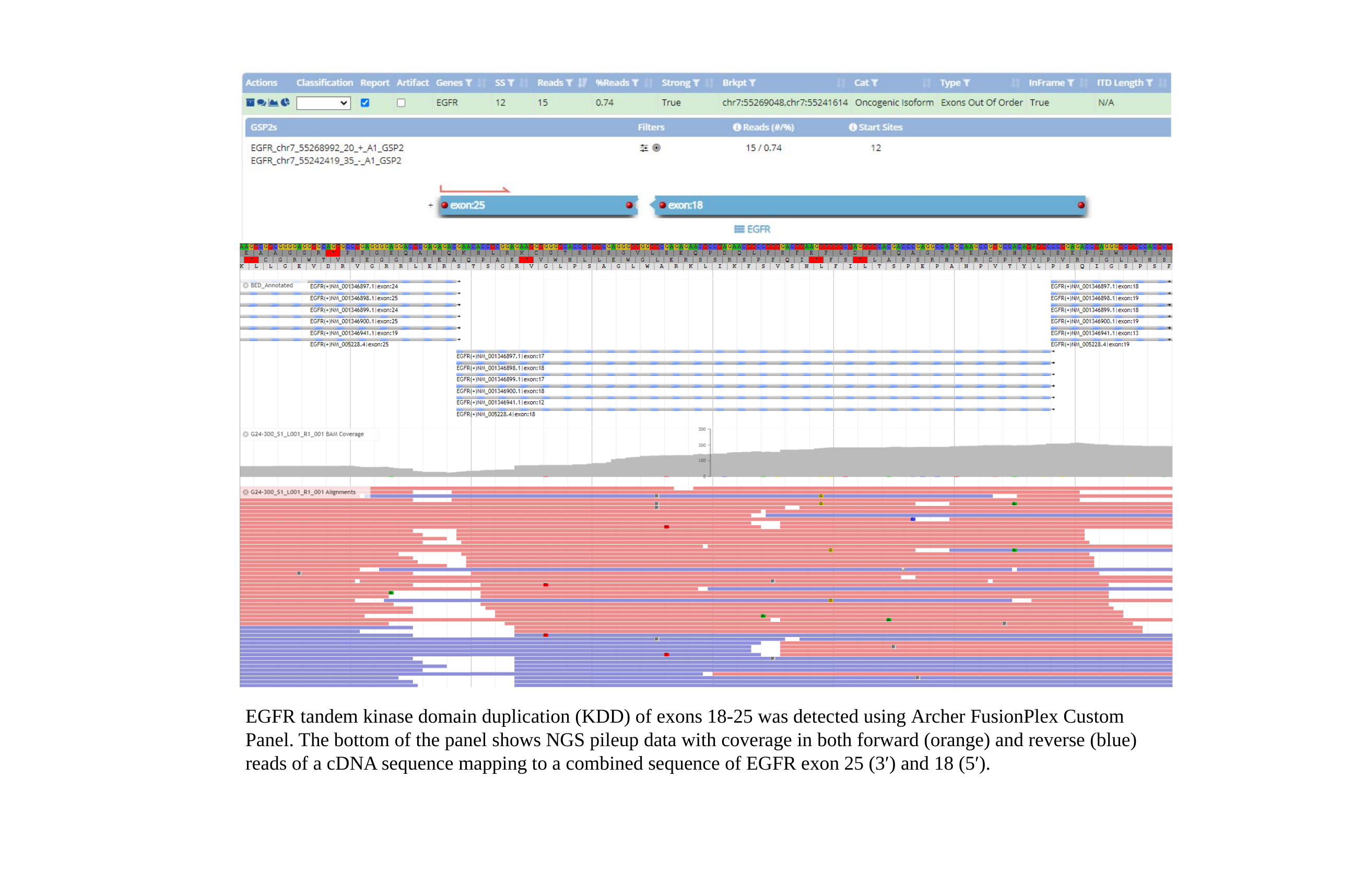

EGFR tandem kinase domain duplication (KDD) of exons 18-25 was detected using Archer FusionPlex Custom Panel. The bottom of the panel shows NGS pileup data with coverage in both forward (orange) and reverse (blue) reads of a cDNA sequence mapping to a combined sequence of EGFR exon 25 (3′) and 18 (5′).
